# Introduced bullfrog facilitates pathogen invasion in the western United States

**DOI:** 10.1371/journal.pone.0188384

**Published:** 2018-04-16

**Authors:** Tiffany A. Yap, Michelle S. Koo, Richard F. Ambrose, Vance T. Vredenburg

**Affiliations:** 1 Institute of the Environment and Sustainability, University of California, Los Angeles, California, United States of America; 2 Museum of Vertebrate Zoology, University of California, Berkeley, California, United States of America; 3 Department of Biology, San Francisco State University, San Francisco, California, United States of America; 4 Department of Environmental Health Sciences, University of California, Los Angeles, California, United States of America; Imperial College Faculty of Medicine, UNITED KINGDOM

## Abstract

*Batrachochytrium dendrobatidis* (*Bd*), a causal agent of the amphibian fungal skin disease chytridiomycosis, has been implicated in the decline and extinction of over 200 species worldwide since the 1970s. Despite almost two decades of research, the history of *Bd* and its global spread is not well understood. However, the spread of the Global Panzootic Lineage of *Bd* (*Bd*-GPL), the lineage associated with amphibian die-offs, has been linked with the American bullfrog (*Rana* [*Aqurana*] *catesbeiana*) and global trade. Interestingly, *R*. *catesbeiana* is native to the eastern U.S., where no *Bd*-related declines have been observed despite *Bd*’s presence since the late 1800s. In contrast *Bd* has been found to have emerged in California and Mexico in the 1960s and 1970s, after which epizootics (*i*.*e*., epidemics in wildlife) ensued. We hypothesize that *Bd*-GPL spread from the eastern U.S. with the introduction of *R*. *catesbeiana* into the western US, resulting in epizootics and declines of native host species. Using museum records, we investigated the historical relationship between *R*. *catesbeiana* and *Bd* invasion in the western US and found that *R*. *catesbeiana* arrived in the same year or prior to *Bd* in most western watersheds that had data for both species, suggesting that *Bd*-GPL may have originated in the eastern US and *R*. *catesbeiana* may have facilitated *Bd* invasion in the western US. To predict areas with greatest suitability for *Bd*, we created a suitability model by integrating habitat suitability and host availability. When we incorporated invasion history with high *Bd* suitability, we found that watersheds with non-native *R*. *catesbeiana* in the mountain ranges of the West Coast have the highest disease risk. These findings shed light on the invasion history and disease dynamics of *Bd* in North America. Targeted historical surveys using archived specimens in natural history collections and present-day field surveys along with more localized, community-level studies, monitoring, and surveillance are needed to further test this hypothesis and grow our understanding of the disease ecology and host-pathogen dynamics of *Bd*.

## Introduction

Chytridiomycosis is an emerging infectious disease primarily caused by the fungal pathogen *Batrachochytrium dendrobatidis* (*Bd*). This pathogen has significantly affected global amphibian biodiversity, infecting over 500 species [1] and causing declines and extinctions in at least 200 species since the 1970s [2–4]. Global trade likely played a role in the current *Bd* pandemic by spreading non-native, infected animals worldwide and exposing naïve populations to *Bd* [3,5–8].

Despite almost two decades of *Bd* research, the history of *Bd* and its global spread is not well understood. However, genomic studies have led to the discovery of multiple *Bd* strains that range in pathogenicity [5,9,10], which provides some insight of the evolutionary history of *Bd*. Different *Bd* lineages have been identified in Brazil, Switzerland, South Africa, and South Korea, where they appear to be in an enzootic state of coexistence with native amphibian populations [5,9,11]. The Global Panzootic Lineage (*Bd*-GPL) is the strain that has been associated with known epizootics (i.e., epidemics in wildlife) [9]. There is evidence that hybridization between *Bd* strains is possible [5], and it has been suggested that hybridization may have led to the origin of *Bd*-GPL [9]. However, genome-wide patterns suggest that mitotic recombination via asexual reproduction is the more likely mechanism to have led to the emergence of *Bd*-GPL [10].

A defining feature of *Bd*-GPL is the presence of specific loss of heterozygosity (LOH) events that may have occurred as recently as 1,000 years ago [10]. The GPL consists of two clades, GPL-1, which is most common in North America, and GPL-2, which is most common in Central and South America [5,12]. Because GPL-2 has additional LOH events that are absent in GPL-1, James et al. [12] hypothesize that GPL-1 is the more ancestral lineage and that GPL-2 diverged some time after it was introduced to Central and South America. Therefore, they conclude that *Bd*-GPL or its parental strain originated in the temperate zone of North America [12].

If *Bd*-GPL originated in North America, then we would expect amphibian populations to be in an enzootic state throughout, as populations never exposed to *Bd* are more likely to be at greater risk of experiencing an epizootic [13–15] compared to those that have existed with the pathogen for an extended amount of time [16–18]. Yet host-pathogen dynamics are not uniform across the continent. The earliest known record of *Bd* is from Illinois in 1888 [18], and pathogen host dynamics east of the Rocky Mountains reflect an enzootic state with no known declines due to *Bd* [18–22]. However, *Bd* epizootics have been documented in the western US (California [15,23], Arizona [24,25], and Colorado [26]) and in Mexico [27]. In addition, several studies have shown that *Bd* invasion occurred in areas of California and Mexico in the 1960s and 1970s [15,27–30].

The eastern US is the native range of the American bullfrog (*Rana* [*Aquarana*] *catesbeiana* [31]), a known *Bd* reservoir [7,32] that is popular in pet and food trade and has been implicated as a vector in global disease spread [3,6,7,33,34]. Rödder et al. [35] showed that *Bd* and *R*. *catesbeiana* have high overlap with their realized niches, further highlighting a link between these two species. The ability of *R*. *catesbeiana* to tolerate *Bd* infection and the lack of *Bd*-related declines in the eastern US, while species in the western US and Mexico have experienced *Bd* epizootics, suggest that *Bd* may have an evolutionary history with the *R*. *catesbeiana* populations in the east. Thus it may be that *Bd*-GPL or its parental strain originated in the eastern US.

We hypothesize that *Bd*-GPL or its predecessor co-evolved with *R*. *catesbeiana* in the eastern US and the introduction of *R*. *catesbeiana* in the western US is an important driver of *Bd* spread. To test this, we investigate the historical presence of *Bd* and *R*. *catesbeiana* in the western US. If indeed *Bd*-GPL originated in the eastern US and *R*. *catesbeiana* played a role in its spread, then we would expect *R*. *catesbeiana* occurrences to coincide with or precede *Bd* occurrences in the western US. We then integrate *Bd* habitat suitability, potential host availability, and the invasion histories of *Bd* and *R*. *catesbeiana* to predict areas where species may have greatest disease risk from *Bd* in North America.

## Materials and methods

### Invasion history

To determine the invasion patterns of *Bd* and *R*. *catesbeiana* outside of *R*. *catesbeiana*’s native range, we compared the historical occurrences of the two species in the watersheds outside the native range of *R*. *catesbeiana* west of the Rocky Mountains. Watershed boundary data (hydrologic unit code 10, HUC10) were obtained from the United States Geological Survey (USGS) [36]. Using 1062 *Bd* positive records in the western US from survey data from the Vredenburg Lab [37] and *Bd*-Maps [38] we identified the earliest record of *Bd* in each watershed. See S1 Table for a summary of *Bd*-positive data from North America. We did the same for *R*. *catesbeiana*, using a total of 2597 occurrence records compiled from several sources: two online repositories that aggregate data from hundreds of natural history collections around the world, VertNet [39] and the Global Biodiversity Information Facility (GBIF [40]), the USGS [37], and any *R*. *catesbeiana* occurrences from the *Bd* survey data. For watersheds that had occurrence data for both species, we identified whether 1) *Bd* was recorded in the same year or prior to *R*. *catesbeiana* or 2) *R*. *catesbeiana* was recorded prior to *Bd*. We also identified watersheds that had only *Bd* occurrences or only *R*. *catesbeiana* occurrences.

### Bd suitability and disease risk

We integrated abiotic and biotic factors to predict the areas in North America with the greatest *Bd* suitability. We first created a presence-only habitat suitability model (HSM) driven by climate and land use factors using Maxent version 3.3.3k [41]. We used 1775 *Bd* occurrence records with 988 unique localities in the North America mainland from the Vredenburg Lab [37] and *Bd*-Maps [38]. See S1 Table for a summary of the *Bd*-positive data used. Boria et al. [42] showed that applying a spatial filter to presence data reduces overfitting; therefore, we applied a 10 arc-minute (~12 km^2^) spatial filter by randomly choosing one *Bd* occurrence site from every 10 arc-minute area using R (dismo [43] and maptools [44] packages). This resulted in 746 *Bd*-positive sites with environmental data for model training. We further minimized sampling bias by restricting background sampling areas to a minimum convex hull around the occurrence points (S1 Fig) [45–49].

We obtained 19 bioclimatic variables from the Worldclim database (http://www.worldclim.org/bioclim), which are a set of interpolated temperature and precipitation conditions based on monthly averages measured at weather stations across the globe from the years 1950 to 2000, latitude, longitude, and elevation [50]. To incorporate land use patterns, we used the global human footprint (HF), which quantifies anthropogenic influences on the terrestrial environment based on land cover, human accessibility, land transformation, human population density, and infrastructure between the years 1993 to 2009 [51]. Some of the bioclimatic variables are highly correlated with each other; therefore, to reduce overfitting of the model due to multicollinearity of the model predictors, we calculated Spearman’s rank correlations (r) among all the variables to determine which variables were highly correlated with each other. When variable pairs had an r^2^ > 0.7, we chose the higher-ranking factor. We then used the resulting subset of 11 environmental variables to create the *Bd* HSM: mean diurnal temperature range (Bio2), temperature seasonality (Bio4), maximum temperature of the warmest month (Bio5) minimum temperature of the coldest month (Bio6), mean temperature of the wettest quarter (Bio8), precipitation of the driest quarter (Bio9), precipitation of the wettest month (Bio13), precipitation of the driest month (Bio14), precipitation of the warmest quarter (Bio18), precipitation of the coldest quarter (Bio19), and human footprint. These variables are biologically relevant to *Bd* and have been shown to perform well in previous *Bd* HSMs [6,12,52–55].

To account for variability and other uncertainties inherent in modeling, we ran 20 replicates for each species using cross-validation: occurrence data were divided into 20 equal-sized folds, or groups, and for each replicate 19 folds were used for model training and one fold was used for model testing. We used 20 replicates because ensemble forecasts have been shown to make models more reliable and robust than single model outputs by accounting for variability [56]. The results were then averaged by Maxent to produce a final probabilistic density function of potentially suitable habitat.

In addition to abiotic factors, biotic factors like host availability or the presence of a vector species are critical for disease maintenance and spread [57]. A pathogen cannot persist without sufficient abundance of susceptible hosts; therefore, the establishment of *Bd* in an area with suitable habitat depends on the presence and abundance of amphibian hosts. Information on host abundance at the community level for a continental assessment is presently not available; however, several studies have shown species richness can be an important factor in *Bd* spread, particularly where a competent reservoir is present [1,6,30,52,58–62]. Thus, in lieu of abundance data, we considered species richness in conjunction with the presence of *R*. *catesbeiana*. To refine our predictions of *Bd* suitability, we calculated the product of the *Bd* HSM and amphibian richness, which was then interpreted at a relative scale.

We estimated amphibian richness in mainland North America by overlaying unique species ranges obtained from the IUCN Red List [63] and AmphibiaWeb [64]. We created a raster dataset in which we tallied the intersecting ranges per grid cell using R (dplyr [65] and raster [66] packages) for total amphibians. Spatial data for range maps compiled by AmphibiaWeb are available at https://github.com/AmphibiaWeb. All range maps are viewable on AmphibiaWeb by species (http://amphibiaweb.org) [64].

The Maxent software calculates multiple threshold values for each model run to aid in model interpretation. Areas with values above these thresholds can be interpreted as a reasonable estimate of a species’ suitable habitat, depending on the quality of the data [67]. Because we modified the Maxent-produced *Bd* HSM by incorporating amphibian richness, for our analyses, we used two thresholds that were proportional to Maxent’s HSM thresholds. To identify areas with general *Bd* suitability (i.e., areas with any likelihood that *Bd* could establish there) we used the minimum training presence logistic threshold, which is the mean of the lowest probability associated with each training point (i.e., true positives) for each of the 20 replicates. To identify areas with high *Bd* suitability (i.e., areas where *Bd* is most likely to establish), we used the most conservative threshold value that gave the lowest predicted area [47], which was the mean value at which training sensitivity and specificity were equal. We then compared the watersheds with species emergence data to the *Bd* suitability model to identify areas with the greatest risk of *Bd* emergence and disease spread.

## Results

### Invasion history

There were 603 out of 6141 western US watersheds with *Bd* and/or *R*. *catesbeiana* occurrence data: 202 had only *Bd* records, 301 had only *R*. *catesbeiana* records, and 100 had records for both species. Records of *R*. *catesbeiana* occurrences documented the same year or prior to *Bd* occurrences were found in 83% (83/100) of the shared watersheds. In nine of the 17 watersheds where *Bd* was documented prior to *R*. *catesbeiana*, *Bd* records preceded *R*. *catesbeiana* by one to seven years. Most (13/17) were adjacent to watersheds where *R*. *catesbeiana* had been documented earlier than the first *Bd* record of the watershed in question (often by decades).

### Bd suitability and disease risk

**Fig 1:**
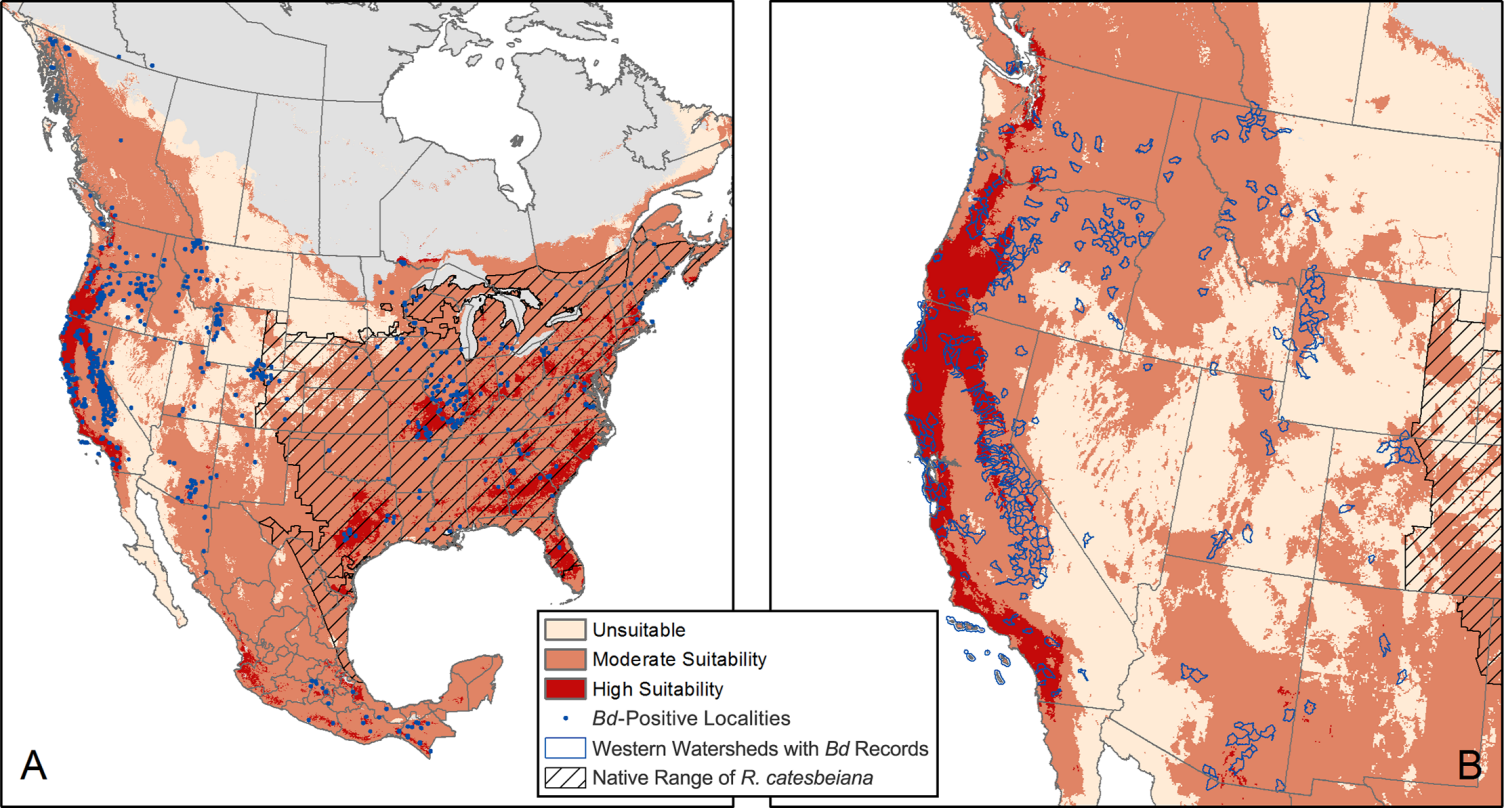
*Bd* suitability and *Bd*-positive localities and watersheds. (A) Areas in North America predicted to have *Bd* suitability (increased intensity in red indicates increased suitability). Blue dots are *Bd* localities. Black hash indicates the native range of *R*. *catesbeiana*. And (B) western US watersheds (in blue) containing *Bd* occurrence records.

Areas predicted to have general *Bd* suitability encompass the eastern half of the US, the Pacific Northwest, portions of mountain ranges in the western US, including the Rocky Mountains and the Sierra Nevada Mountains, and most of Mexico (Fig 1A). The areas deemed unsuitable include the arid and semi-arid regions in the Great Plains and the desert. Approximately 95% (940/988) of *Bd*-positive localities and 94% (2567/2743) of *R*. *catesbeiana* localities were found to be in predicted general *Bd* suitability areas (Fig 1A). All but two western US watersheds that had *Bd* occurrence records overlapped with general *Bd* suitability areas (300/302) (Fig 1B). See Supplemental S2 Fig for a breakdown of the different watersheds types (i.e., where *Bd* was recorded before *R*. *catesbeiana* [17/17], where *R*. *catesbeiana* was recorded in the same year or before *Bd* [82/83], and where only *Bd* has been recorded [201/202]). Most of the watersheds where *R*. *catesbeiana* were recorded overlapped with general *Bd* suitability areas (95% [286/301] of watersheds where only *R*. *catesbeiana* were recorded and 96% [385/401] of all watersheds in the western US where *R*. *catesbeiana* has been recorded; S2 Fig).

**Fig 2:**
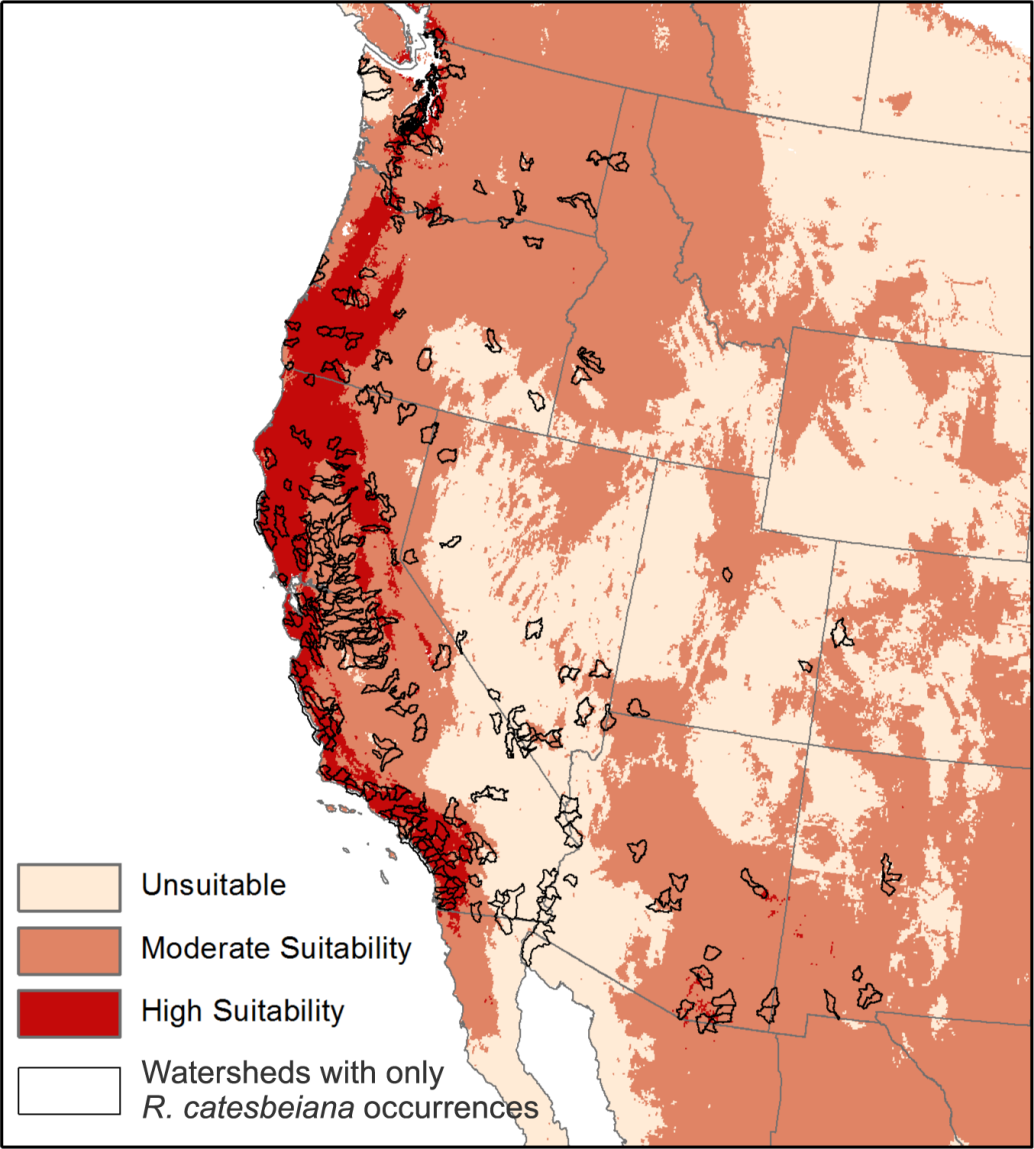
High *Bd* risk. Areas in North America predicted to have high *Bd* suitability (deep red) and western US watersheds containing only *R*. *catesbeiana* occurrence records (in black). Overlapping areas are predicted to have the highest risk of *Bd* emergence and outbreaks.

Areas predicted to have high *Bd* suitability include mountain ranges along the West Coast of the US, the highlands of Central Mexico, the Coastal Plains of the Southeast US, and the Ozark Plateau (Fig 1). Of the 301 watersheds where only *R*. *catesbeiana* has been recorded, 173 (57%) overlap with high *Bd* suitability areas (Fig 2). These areas are predicted to have the greatest risk of *Bd* outbreaks.

## Discussion

Chytridiomycosis has severely impacted amphibian biodiversity globally for decades, with *Bd* infecting hundreds of amphibian species worldwide [1–4]. Although our knowledge of *Bd* is incomplete, our understanding of this complex host-pathogen system continues to grow through a variety of ongoing studies of its biology and disease dynamics. By investigating the historical emergence of *Bd* and *R*. *catesbeiana* in the western US, we uncover evidence of a historical relationship between the invasion of *Bd* and the introduction of *R*. *catesbeiana*. We show that *R*. *catesbeiana* occurred prior to *Bd* emergence in most of the shared watersheds, which supports Huss et al. [34] and suggests that the introduction of *R*. *catesbeiana* may have played a significant role in spreading *Bd* to naïve amphibian populations in the western US.

The *Bd* suitability model is generally similar to those from previous studies [6,12,52,68,69]. Our model indicates that there is *Bd* suitability in *R*. *catesbeiana*’s native range in the eastern US (Fig 1A); however, this likely reflects the high environmental and host suitability for *Bd* in a region of endemism rather than the threat of disease from *Bd* to naïve amphibian populations, given that no *Bd*-related declines have been documented in the eastern US despite *Bd*’s presence [18–22]. In addition, 94% of *R*. *catesbeiana* occurrence data and 96% of western watersheds where *R*. *catesbeiana* has been recorded were within general *Bd* suitability areas (Fig 2). This further highlights that *Bd* and *R*. *catesbeiana* have high niche overlap [35]. While these results are correlational, they support a growing number of studies that link *Bd* emergence with the presence of introduced *R*. *catesbeiana* [3,6–8,34,70–74] and are consistent with the hypothesis that *Bd*-GPL (or its preceding strain) may have co-evolved with *R*. *catesbeiana* in the eastern US.

If we consider the historical link between the emergence of *Bd* and *R*. *catesbeiana* in the western US, we can identify the areas where native species are at greatest risk of disease. Thus, species that occur in watersheds where only *R*. *catesbeiana* has been documented that overlap with areas predicted to have high *Bd* suitability (Fig 2) could have increased risk of *Bd* emergence. The presence of a reservoir host like *R*. *catesbeiana* (or others [75]) could lead to local population declines due to disease, even well beyond the initial emergence of *Bd* [60]. High risk areas include many watersheds in the Cascade Range in the Pacific Northwest, the California coastal ranges, and the Sierra Nevada Mountains, all areas where native amphibians have declined [15,76–78].

Improving our understanding of the distribution and spread of *Bd* enhances our knowledge of *Bd*’s history and provides vital information for amphibian conservation and management strategies. Areas identified as having the highest risk of disease should be prioritized for both historical surveys using archived specimens in natural history collections [27] and present-day field surveys to investigate our hypothesis that the introduction of *R*. *catesbeiana* facilitates *Bd* spread. In addition, proactive management efforts could minimize disease spread in these areas [79].

It is important to consider that *Bd* is not the only disease threat to amphibian diversity. *Batrachochytrium salamandrivorans* (*Bsal*), a second, more recently discovered chytrid pathogen that has not yet been found in North America [19,20], poses another serious threat to North American amphibians [80,81]. The continuous spread of *Bd* threatens remaining uninfected populations, and the introduction of *Bsal* could compound the likelihood of more amphibian epizootics.

The most disruptive effect of a single pathogen infection is death; however, surviving species could incur sub-lethal effects from infection (or from fighting off infection) that could suppress immune defenses against other stressors [82–86], such as a second deadly pathogen. This could at least partially explain the *Bsal*-induced mass mortalities of European fire salamanders (*Salamandra salamandra*) in the Netherlands, where *Bd* was co-existing with local amphibian populations in an enzootic state [87,88]. Thus, species surviving in areas where *Bd* is already present could be more vulnerable to *Bsal* exposure, and the introduction of *Bsal* could result in disease outbreaks.

Alternatively, species or populations previously exposed to a similar pathogen might benefit from direct competition between pathogens or cross immunity, in which a host acquires immunity or partial immunity to one pathogen because of previous infection from either the same pathogen or a closely related pathogen [89]. This could potentially explain the lack of *Bd*-related declines in areas where both an endemic strain of *Bd* and *Bd*-GPL are present, such as in Brazil, South Africa, Switzerland, and South Korea [11,17,90]. Perhaps native *Bd* strains are able to outcompete introduced strains like *Bd*-GPL, or the evolutionary history with endemic *Bd* lineages has primed local amphibian populations to be able to resist or tolerate infection from *Bd*-GPL [5,11]. If previous exposure to *Bd* leads to either *Bd* outcompeting *Bsal* for hosts or cross immunity to *Bsal*, then persisting populations in areas where *Bd* is in an enzootic state may be safeguarded against *Bsal* infections, and amphibians in areas where *Bd* is more recently established or where neither *Bd* nor *Bsal* currently occur may have the highest disease risk. More studies are needed to understand the potential interactions of these pathogens.

## Conclusion

We investigated the invasion history of *R*. *catesbeiana* and *Bd* in the western US and found a pattern of *Bd* dynamics consistent with the hypothesis that invasion of *R*. *catesbeiana* facilitated *Bd* spread in western North America. This supports the hypothesis that *Bd*-GPL (or its preceding strain) may have originated in the eastern US. We found that the historical presence of *R*. *catesbeiana* outside its native range was highly correlated with emergence of *Bd* in areas predicted to be suitable for *Bd*. We identified areas of increased disease risk by integrating environmental suitability, host availability, and invasion history to provide guidance for conservation and management efforts. Targeted historical surveys using archived specimens in natural history collections and present day field surveys could help test our hypotheses and further our understanding of the spatiotemporal distribution of *Bd*. More localized, community-level studies that include monitoring and surveillance are needed to further our understanding of the disease ecology and host-pathogen dynamics of chytridiomycosis. Species susceptibility studies could lead to the identification of the potential interactions between *Bd* and *Bsal* and the discovery of potential defenses against disease.

## Supporting information

S1 Fig Training points and background sampling areas for the *Bd* habitat suitability model. (TIF)

S2 Fig Invaded western US watersheds compared to *Bd* suitability. (A) Shared watersheds where *Bd* was recorded prior to *R*. *catesbeiana* (blue) and where *R*. *catesbeiana* was recorded in the same year or prior to *Bd* (black), (B) watersheds where only *Bd* has been recorded, and (C) watersheds where only *R*. *catesbeiana* has been recorded.
(TIF)

S1 Table Summary of *Bd*-positive records used for the *Bd* HSM. Species and decades in which *Bd* was detected in the wild in North America.
(XLSX)
