# Supplementary figures and images for: Introduced bullfrog facilitates pathogen invasion in the western United States
Source: PLoS One. 2018 Apr 16;13(4):e0188384. doi: 10.1371/journal.pone.0188384 (PMC5901863; doi:10.1371/journal.pone.0188384)

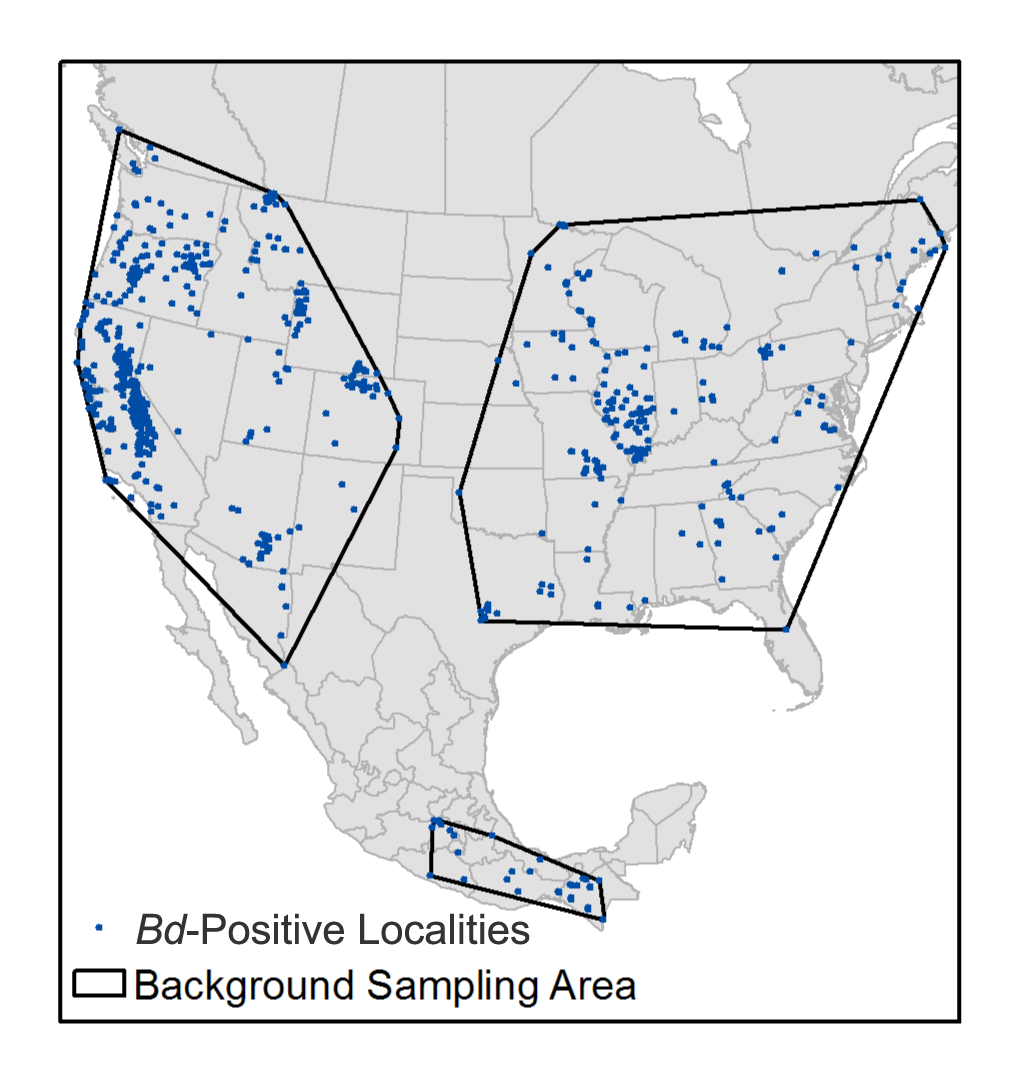

Supplement: S1 Fig — (TIF) [file pone.0188384.s001.tif]

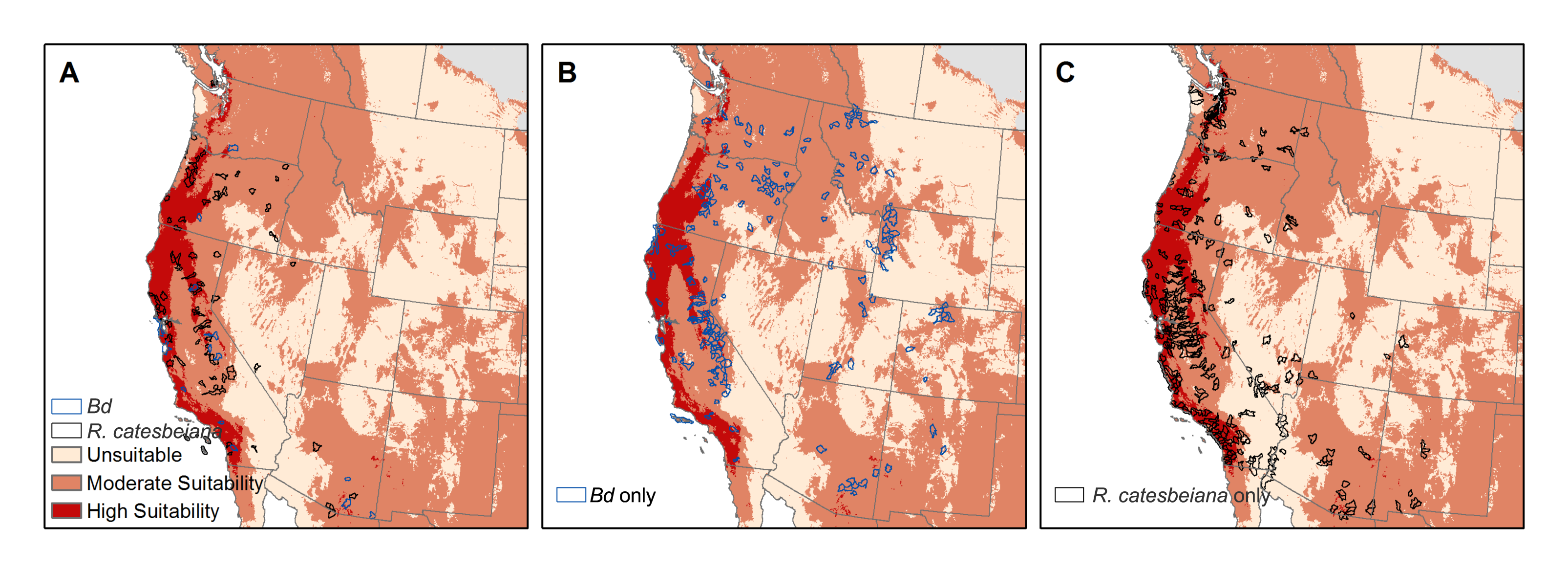

Supplement: S2 Fig — (A) Shared watersheds where Bd was recorded prior to R. catesbeiana (blue) and where R. catesbeiana was recorded in the same year or prior to Bd (black), (B) watersheds where only Bd has been recorded, and (C) watersheds where only R. catesbeiana has been recorded. (TIF) [file pone.0188384.s002.tif]
